# Obese elderly patients with hip fractures may have better survival outcomes after surgery

**DOI:** 10.1007/s00402-023-04787-0

**Published:** 2023-02-09

**Authors:** Hao Wang, Liping Pan, Baoqiang Li, Taiguo Ning, Guanghua Liang, Yongping Cao

**Affiliations:** 1 Department of Orthopedics, Peking University First Hospital, No. 8 Xishiku Street, XiCheng District, Beijing, 100034 China; 2 Department of Orthopedics, Beijing Chao-Yang Hospital, 8 Gongren Tiyuchang Nanlu, Chaoyang District, Beijing, 100020 China

**Keywords:** BMI, Obesity, Complications, Hip fracture, Mortality, AKI

## Abstract

**Background:**

In recent years, there has been an increasing amount of research on the “obesity paradox”. So our primary objective was to explore whether this phenomenon exists in our study, and secondary objective was to determine the effect of body mass index (BMI) on major complications, and the incidence of acute kidney injury (AKI) after hip fracture surgery after controlling for confounding factors.

**Methods:**

We included patients over 70 years old with hip fracture who were admitted to the Department of Orthopedics, Peking University First Hospital between 2015 and 2021. Patients were classified as underweight (UW, < 18.5 kg/m^2^), normal weight (NW, 18.5–24.9 kg/m^2^), overweight (OW, 25.0–29.9 kg/m^2^) and obese (OB, ≥ 30.0 kg/m^2^). We analyzed demographic characteristics, operation information and postoperative outcomes. Using multivariate regression with normal-weight patients as the reference, we determined the odds of 1-year mortality, major complications, and AKI by BMI category.

**Results:**

A total of 644 patients were included. Nine percent of patients died after 1 year, 18% had major postoperative complications, and 12% had AKI. There was a U-shaped relationship between BMI and the rates of major complications or AKI. However, there was a linear decreasing relationship between 1-year mortality and BMI. After controlling for confounding factors, multivariate regression analysis showed that the risk of 1-year mortality after surgery was 2.24 times higher in underweight patients than in normal-weight patients (*P < *0.05, OR: 2.24, 95% CI 1.14–4.42). Compared with normal-weight patients, underweight patients had a 2.07 times increased risk of major complications (*P < *0.05, OR 2.07, 95% CI 1.21–3.55), and the risk of major complications in obese patients was 2.57 times higher than that in normal-weight patients (*P < *0.05, OR 2.57, 95% CI 1.09–6.09). Compared with normal-weight, underweight patients had a 2.18 times increased risk of AKI (*P < *0.05, OR 2.18, 95% CI 1.17–4.05).

**Conclusions:**

The 1-year mortality risk of patients with higher BMI was significantly reduced. Besides, compared with normal-weight patients, underweight patients and obese patients have a higher risk of major complications; low-weight and obese patients are at higher risk for AKI.

## Introduction

Hip fracture is associated with excess mortality (over and above mortality rates in non-hip fracture/community control populations) during the first year after fracture; the values range from 8.4 to 36% [2]. With modest assumptions concerning secular trends, the number of hip fractures may range between 7.3 and 21.3 million by 2050. Major demographic changes will occur in Asia [14]. In China, the absolute number of hip fractures and associated medical costs for hospitalization have increased rapidly because of aging in the population [37].

In today's world, an increasing number of people are overweight or obese, accounting for one-third of the global population, and obesity has become a recognized chronic disease [10]. Obesity not only causes inconvenience in activities but is also a major factor in chronic diseases such as hypertension, diabetes and coronary heart disease [5]. Although obesity can cause a variety of adverse complications, in recent years, some researchers have found that obesity may lead to better clinical outcomes for some diseases, which seems to contradict people's understanding of obesity and is called the “obesity paradox.” Although the pathophysiology of the “obesity paradox” is still unclear, it can be hypothesized that fat is the first energy substance consumed after traumatic stress, followed by high energy consumption in obese patients. Therefore, the consumption of fat greatly reduces the consumption of important proteins and avoids decrease in immunity and loss of nutrients in obese patients [15, 17]. According to a review in 2017, most studies show a positive relationship between BMI and bone mineral density(BMD) [33]. Other systematic reviews have shown that obese patients are less likely to die after acute coronary syndromes and heart failure than normal-weight patients [23]. Some studies have shown that obese or overweight patients have lower mortality or better clinical outcomes after a hip fracture than normal-weight patients [22, 29]. However, these studies did not adjust for the influence of confounding factors such as age, sex, CCI, surgical type and surgical delay time on BMI. In this study, the effects of age, sex, CCI, surgical type and surgical delay time on BMI were adjusted to explore the effects of BMI on 1-year postoperative mortality and postoperative complications.

## Methods

### Source of patients, inclusion criteria, and exclusion criteria

**Fig. 1:**
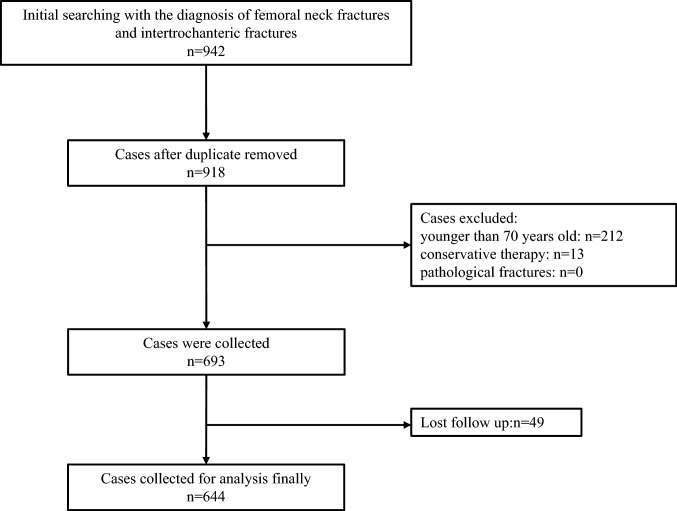
The flowchart of screening patients

This was a single-center retrospective study that collected data on hip fracture patients at Peking University First Hospital between 2015 and 2021. The inclusion criteria were as follows: (1) over 70 years old; (2) hip fractures including femoral neck fractures and intertrochanteric fractures; (3) surgical procedures including internal fixation, hemiarthroplasty (HHA), and total hip arthroplasty (THA). The exclusion criteria were as follows: (1) under 70 years old; (2) pathological fractures; (3) conservative treatment of hip fracture; (4) revision surgery; (5) The patients had multiple traumas and underwent other surgeries in addition to hip surgery. Their demographic characteristics and relevant perioperative information were collected. A total of 942 patients with femoral neck fracture and intertrochanteric fracture were initially screened. A total of 918 patients were obtained after removing duplicate patients by checking the medical record number, and 693 patients were obtained after excluding patients younger than 70 years, conservative treatment and pathological fractures. We followed up the patients by telephone for 1 year. If the patients died within 1 year, the time of death was recorded. Forty-nine patients were lost to follow-up, and a total of 644 patients were finally included in the study. The specific inclusion process of patients is shown in Fig. 1. This study was a retrospective study, which did not have any adverse influence on patients, and we have guaranteed the confidentiality of the patients' medical information. The study was approved by the Ethics Committee of Peking University First Hospital (NO. 2021-432).

### Data extraction

We used the World Health Organization BMI classification criteria and divided BMI into four levels: underweight (UW) (BMI < 18.5 kg/m^2^), normal weight (NW) (BMI 18.5–24.9 kg/m^2^), overweight (OW) (BMI 25.0–29.9 kg/m^2^) and obese (OB) (BMI ≥ 30.0 kg/m^2^) [1]. With BMI as the classification standard, we collected data on each patient's height, weight, age, sex, preoperative comorbidities, fracture type, operation delay time, ASA, CCI, and preoperative anemia as demographic characteristics before surgery as shown in Table 1. The classification of anemia is based on the WHO classification standards: mild anemia: hemoglobin(Hb) of 90–120 g/L for male and 90–110 g/L for female; Moderate anemia: Hb of 60–90 g/L; Severe anemia: Hb of 30–60 g/L; Very severe anemia: Hb less than 30 g/L. The information on the operation is shown in Table 2, including operation duration, surgery type, anesthesia method and hypotension of more than 15 min. The postoperative conditions are shown in Table 3, including blood transfusion, postoperative anemia, major complications (life-threatening), minor complications (not life-threatening), and mortality at 30 days, 3 months, and 1 year after surgery. Except for postoperative mortality, the other included variables were from the surgical, nursing and anesthesia data in the medical record system.

**Table 1:** Demographic characteristics of patients after hip fracture surgery and the proportion in different BMI classification

	Total (*n = *644)	UW^a^ (*n = *105)	NW^a^ (*n = *362)	OW^a^ (*n = *126)	OB^a^ (*n = *30)	*P* value
Sex, *n* (%)						0.069
Male	174 (27)	22 (21)	111 (31)	35 (28)	4 (13)	
Female	470 (73)	83 (79)	251 (69)	91 (72)	26 (87)	
Age (mean ± SD)	81.66 ± 6.15	83.60 ± 5.93	82.09 ± 6.34	80.20 ± 6.24	81.97 ± 5.51	0.001*
Fracture type, *n* (%)						0.292
Femoral neck fracture	377 (59)	53 (50)	217 (60)	77 (61)	19 (63)	
Intertrochanterric fracture	267 (41)	52 (50)	145 (40)	49 (39)	11 (37)	
Surgery delay, *n* (%)						0.625
Less than 48 h	297 (46)	50 (48)	164 (45)	62 (49)	11 (37)	
More than 48 h	347 (54)	55 (52)	198 (55)	64 (51)	19 (63)	
ASA, *n* (%)						0.937
2	239 (37)	41 (40)	137 (38)	45 (36)	10 (33)	
3	362 (56)	54 (52)	204 (56)	72 (57)	18 (60)	
4	38 (6)	8 (8)	20 (6)	8 (6)	1 (3)	
CCI, *n* (%)						0.738
Less than 4	221 (34)	44 (42)	124 (34)	36 (29)	11 (37)	
5	200 (31)	27 (26)	111 (31)	46 (37)	10 (33)	
6	115 (18)	16 (15)	67 (19)	22 (17)	5 (17)	
7	61 (9)	10 (10)	33 (9)	13 (10)	4 (13)	
More than 8	47 (7)	8 (7)	27 (7)	9 (7)	0	
Preoperative anemia, *n* (%)	197 (31)	51 (48)	104 (28)	30 (24)	5 (17)	< 0.001*
No anemia	445 (69)	53 (50)	257 (71)	96 (76)	25 (83)	
Mild anemia	157 (24)	40 (38)	81 (22)	27 (21)	5 (17)	
Moderate anemia	40 (6)	11 (12)	23 (6)	3 (2)	0	
Severe anemia	0	0	0	0	0	
Extremely severe anemia	0	0	0	0	0	
Comorbidities, *n* (%)	532 (83)	77 (73)	307 (85)	109 (87)	24 (80)	0.028*
Heart diease	251 (39)	33 (31)	142 (39)	56 (44)	15 (50)	0.136
Cerebrovascular disease	171 (27)	18 (17)	103 (28)	36 (29)	6 (20)	0.095
Hypertension	401 (62)	48 (46)	225 (62)	98 (78)	22 (73)	< 0.001*
Diabetes mellitus	190 (30)	16 (15)	115 (32)	37 (29)	13 (43)	0.003*
Respiratory disease	87 (14)	20 (19)	43 (12)	18 (14)	4 (13)	0.307
Kidney disease	28 (4)	4 (4)	16 (4)	8 (6)	0	0.569

*Denotes statistical significance

^a^Underweight (UW) (BMI < 18.5 kg/m^2^), normal weight (NW) (BMI 18.5–24.9 kg/m^2^), overweight (OW) (BMI 25.0–29.9 kg/m^2^), obese (OB) (BMI ≥ 30.0 kg/m^2^)

**Table 2:** Surgery-related information and proportion according to BMI classification after hip fracture

	Total (*n = *644)	UW^a^ (*n = *105)	NW^a^ (*n = *362)	OW^a^ (*n = *126)	OB^a^ (*n = *30)	*P* value
Operation duration, *n* (%)						0.039*
Less than 1 h	210 (33)	44 (42)	125 (35)	29 (23)	8 (27)	
1–2 h	371 (57)	51 (48)	210 (58)	79 (63)	18 (60)	
More than 2 h	62 (10)	10 (10)	27 (7)	17 (13)	4 (13)	
Surgery type, *n* (%)						0.633
Internal fixation	294 (46)	53 (50)	160 (44)	57 (45)	13 (43)	
HHA	330 (51)	50 (48)	191 (53)	62 (49)	17 (57)	
THA	20 (3)	2 (2)	11 (3)	7 (6)	0	
Anesthesia type, *n* (%)						0.008*
Intravertebral anesthesia	446 (69)	72 (69)	268 (74)	77 (61)	16 (53)	
General anesthesia	197 (31)	33 (31)	93 (26)	49 (39)	14 (47)	
Hypotension 90 mmHg(15 min or more), *n* (%)	45 (7)	6 (6)	23 (6)	11 (9)	3 (10)	0.681

*Denotes statistical significance

^a^Underweight (UW) (BMI < 18 5 kg/m^2^), normal weight (NW) (BMI 18.5–24.9 kg/m^2^), overweight (OW) (BMI 25.0–29.9 kg/m^2^), obese (OB) (BMI ≥ 30.0 kg/m^2^)

**Table 3:** Postoperative information and proportion in different BMI classifications after hip fracture

	Total (*n = *644)	UW^a^ (*n = *105)	NW^a^ (*n = *362)	OW^a^ (*n = *126)	OB^a^ (*n = *30)	*P* value
Transfusion, *n* (%)						0.011*
No	362 (56)	46 (44)	207 (57)	76 (60)	22 (73)	
Yes	282 (44)	59 (56)	155 (43)	50 (40)	8 (27)	
Postoperative anemia, *n* (%)	488 (76)	84 (80)	277 (77)	90 (71)	23 (77)	0.277
No anemia	153 (24)	20 (19)	83 (23)	36 (29)	7 (23)	
Mild anemia	382 (59)	62 (59)	213 (59)	76 (60)	19 (63)	
Moderate anemia	102 (16)	22 (21)	61 (17)	14 (11)	3 (10)	
Severe anemia	4 (1)	0	3 (1)	0	1 (3)	
Extremely severe anemia	0	0	0	0	0	
30-day mortality, *n* (%)	11 (2)	4 (4)	4 (1)	3 (2)	0	0.239
3-mouth mortality, *n* (%)	30 (5)	9 (9)	14 (4)	5 (4)	1 (3)	0.240
1-year mortality, *n* (%)	56 (9)	17 (16)	31 (9)	6 (5)	1 (3)	0.013*
Complications, *n* (%)	160 (25)	30 (29)	82 (23)	34 (27)	9 (30)	0.485
Major complications(Life-threatening), *n* (%)	119 (18)	29 (28)	56 (15)	22 (17)	9 (30)	0.014*
Acute renal failure	78 (12)	20 (19)	34 (9)	16 (13)	6 (20)	0.029*
Pulmonary embolism	6 (1)	2 (2)	4 (1)	0	0	0.516
Myocardial infarction	14 (2)	3 (3)	8 (2)	3 (2)	0	0.964
Stroke	14 (2)	4 (4)	5 (1)	3 (2)	2 (7)	0.108
Arrhythmia	22 (3)	3 (3)	12 (3)	4 (3)	2 (7)	0.669
Minor complications(No life-threatening), *n* (%)	108 (17)	23 (22)	54 (15)	23 (18)	5 (17)	0.383
Pneumonia	24 (4)	6 (6)	9 (2)	4 (3)	3 (10)	0.081
Pulmonary insufficiency	17 (3)	4 (4)	8 (2)	4 (3)	1 (3)	0.618
Other site infections	9 (1)	0	4 (1)	4 (3)	1 (3)	0.112
Heart failure	28 (4)	7 (7)	14 (4)	6 (5)	1(3)	0.611
Delirium	45 (7)	8 (8)	29 (8)	5 (4)	2 (7)	0.499

*Denotes statistical significance

^a^Underweight (UW) (BMI < 18.5 kg/m^2^), normal weight (NW) (BMI 18.5–24.9 kg/m^2^), overweight (OW) (BMI 25.0–29.9 kg/m^2^), obese (OB) (BMI ≥ 30.0 kg/m^2^)

### Surgery

Patients were diagnosed by the anteroposterior projection of bilateral hip joints and the lateral projection of unilateral hip joint; operation methods were roughly divided equally into internal fixation, HHA and THA, with fracture type, age, and physical condition taken into account. Postoperative X-ray was used to ensure the suitable position of the prosthesis, plate or screws. The operations were performed by senior orthopedic professors. The operative time was defined as the time from skin incision to skin suturing.

### Data processing

Continuous variables are expressed as the mean ± SD, and categorical variables are expressed as percentages. The differences in continuous variables with normal distributions were analyzed by *t* test or ANOVA test. The Kruskal‒Wallis test or Mann–Whitney test was used to analyze the differences in continuous variables that did not fit the normal distribution. The differences in categorical variables were analyzed by the chi-square test.

Logistic regression analysis was used to analyze underweight, overweight, obese patients’ 1-year mortality after surgery, and postoperative complications to determine whether there was a significant correlation compared with normal-weight patients, controlling for confounding factors such as age, sex, CCI, surgery type, and operation delay time and determining the OR and 95% confidence intervals (CIs). These controlled covariates were included in the regression models on the basis of a priori evidence indicating their relationships with morbidity and death [26, 36]. The significance level was defined as *P < *0.05. SPSS (Version 25, IBM SPSS Statistics for Windows, Armonk, NY, USA) was used for data analysis.

## Results

A total of 644 patients were included in the study, of which 16% were underweight, 56% were normal weight, 20% were overweight, and 8% were obese. There were 27% male patients and 73% female patients. For each level of BMI, there was a higher proportion of women than men (*P < *0.001). The average age of the patients was 81.66 ± 6.15 years. ANOVA test showed that overweight patients were younger than underweight patients and normal-weight patients (*P < *0.001). There were no significant differences in fracture type, surgical method, ASA score or CCI score among the different levels of BMI (*P > *0.05). In our study, preoperative anemia was divided into four categories: no anemia, mild anemia, moderate anemia, severe anemia and very severe anemia. Among these categories, patients with anemia accounted for 30% before the operation, but no patients with severe anemia or very severe anemia were found in our study. For the patients without anemia before surgery, there was a linear increasing trend with increase in BMI. The proportion of patients with mild and moderate anemia decreased with BMI (*P < *0.001); the correlation coefficient between preoperative anemia and BMI was 0.198 (*P < *0.05). The proportion of preoperative comorbidities in different BMI grades was different (*P < *0.05). Patients with hypertension accounted for up to 73% of overweight patients, a significantly higher proportion than in the underweight and normal weight groups (*P < *0.001), and the correlation coefficient between BMI and hypertension was 0.203 (*P < *0.05). In addition, the distribution proportion of preoperative patients with diabetes was also significantly different in groups of different BMI grades (*P = *0.001). However, there was no significant difference in the proportion of heart disease, cerebrovascular disease, respiratory disease and kidney disease in BMI (*P > *0.05).

The operation time was defined as the time from skin incision to skin suturing and was classified into three categories: less than 1 h, 1–2 h, and more than 2 h. Statistical analysis showed that the duration of surgery was associated with BMI (*P < *0.05, *r = *0.145). The proportion of patients with an operation time less than 1 h was significantly higher in the group of underweight patients than among overweight and obese patients. Briefly, the greater the weight, the longer the operation. There were significant differences in the distribution of anesthesia methods according to BMI (*P < *0.05); underweight patients were more likely to undergo intraspinal anesthesia, while obese patients were more likely to undergo general anesthesia. There was no significant difference in BMI distribution between the surgical method or the occurrence of intraoperative hypotension lasting over 15 min.

**Fig. 2:**
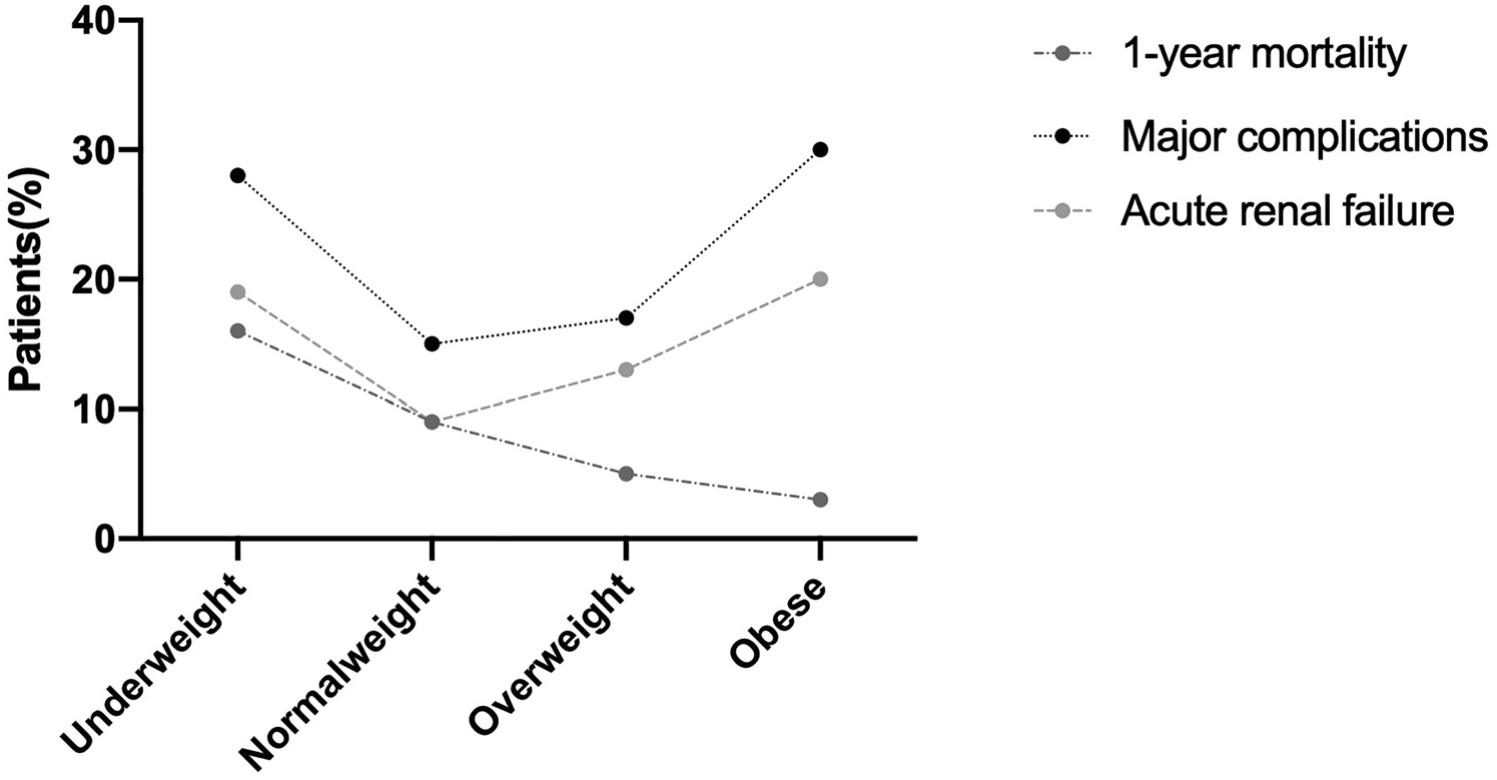
Percentages of 644 patients who experienced complications or death within 1 year after hip fracture surgery by BMI category

Compared with the underweight, the groups of overweight and obese patients revealed lower proportions of perioperative blood transfusions (*P < *0.05). After 1 year of follow-up, we found that underweight patients were significantly more likely to die 1 year after surgery than overweight and obese patients (*P < *0.05), and there was a linear decreasing relationship with BMI. However, there was no significant difference in mortality between patients with different BMIs at 30 days and 3 months after surgery. Analysis of the rates of major complications and AKI revealed a U-shaped relationship with BMI (*P < *0.05) as shown in Fig. 2.

**Fig. 3:**
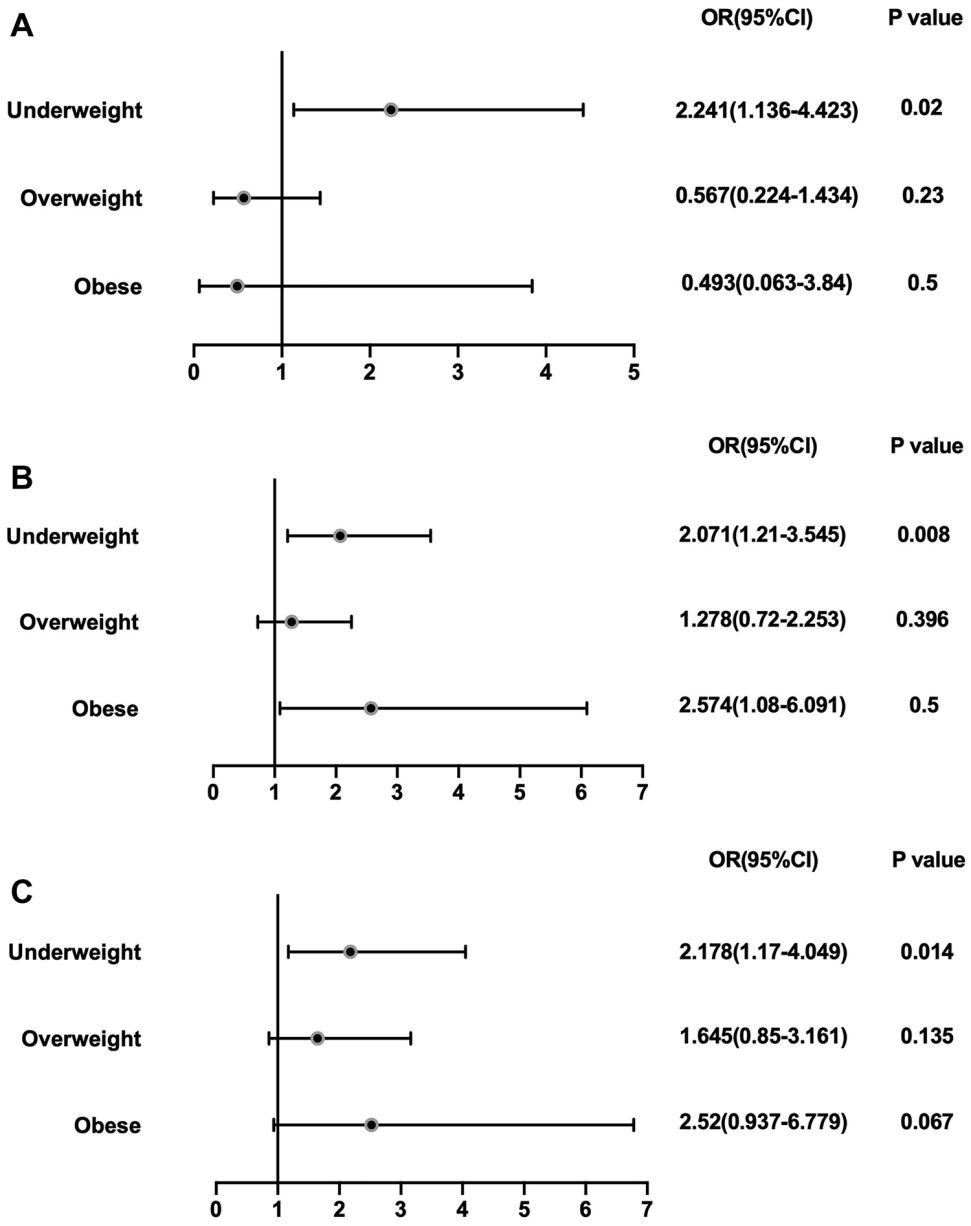
Forest plots showing odds ratios and 95% confidence intervals for patients experiencing **A**) 1-year motorlity, **B**) major complication, **C**) acute renal failure within 1 year after hip fracture surgery compare to normal weight

To explore whether BMI was an independent risk factor for 1-year mortality and postoperative complications, we used normal weight as the reference and performed logistic regression analysis after controlling for confounding factors such as age, sex, surgery type, operation delay time and CCI. The results are shown in Table 4 and Fig. 3. Logistic regression showed that if normal weight was used as the reference, underweight patients had a 2.24-fold increased risk of death 1 year after surgery (*P < *0.05, OR 2.24, 95% CI 1.14–4.42). However, there was no significant difference in 1-year mortality between overweight and obese patients compared with normal-weight patients. Logistic regression showed that underweight patients had a 2.07-fold increased risk of major postoperative complications compared with normal-weight patients (*P < *0.05, OR 2.07, 95% CI 1.21–3.55). Similarly, obese patients had a 2.57-fold increased risk of major postoperative complications compared with normal-weight patients (*P < *0.05, OR 2.57, 95% CI 1.09–6.09). Logistic regression showed that underweight patients had a 2.18-fold increased risk of AKI (*P < *0.05, OR 2.18, 95% CI 1.17–4.05).

**Table 4:** Adjusted Odds of major complications, acute renal failure and mortality within 1 year after hip fracture surgery by BMI Category

BMI^a^ categories	1-year mortality		Major complications		Acute renal failure	
	OR(95%)	*P*	OR(95%)	*P*	OR(95%)	*P*
NW	Referent	0.025	Referent	0.02	Referent	0.043
UW	2.24 (1. 14–4. 42)	0.02	2.07 (1. 21–3. 55)	0.008	2.18 (1. 17–4. 05)	0.014
OW	0.57 (0. 22–1. 43)	0.23	1.28 (0. 73–2. 25)	0.396	1.65 (0. 86–3. 16)	0.135
OB	0.49 (0. 06–3. 84)	0.5	2.57 (1. 09–6. 09)	0.031	2.52 (0. 94–6. 78)	0.067

Adjusted for age, sex, surgery type, CCI and surgery delay

^a^BMI categories are defined as follows: underweight (UW) (BMI < 18. 5 kg/m^2^), normal weight (NW) (BMI 18.5–24.9 kg/m^2^), overweight (OW) (BMI 25.0–29.9 kg/m^2^), obese (OB) (BMI ≥ 30.0 kg/m^2^)

## Discussion

At present, the proportion of obese patients in the world population is gradually increasing, and obesity will lead to diabetes, cerebrovascular disease, heart disease, hypertension and other chronic diseases [16]. There are similar findings in our study. As shown in Table 1, the probability of these chronic diseases was significantly higher in overweight and obese patients than in underweight and normal-weight patients. As a result, the annual cost of treating chronic diseases caused by obesity is also increasing, accounting for as much as 10% of healthcare costs in the United States[13]. A hip fracture is a catastrophic condition for elderly individuals, colloquially known as the “last fracture of life.” Elderly patients need to rest in bed after hip fracture, which will not only lead to the gradual weakness but also magnify the impact of combined chronic diseases and increase the mortality and disability rate for such patients[7, 14, 19]. In contrast to studies that suggest obesity is a harmful factor, some studies have shown that a higher BMI protects bone health and reduces the risk of death after hip fracture surgery [31].

In our study, underweight patients had a significantly higher risk of death 1 year after surgery than normal-weight patients after controlling for age, sex, CCI, surgery type and operation delay time; the risk was 2.24 times higher than that of normal-weight patients. However, there was no significant difference in the risk of death for overweight and obese patients 1 year after surgery compared with normal-weight patients. In addition, the proportion of the number of deaths 1 year after surgery at different BMI levels gradually decreased, which indicated that BMI was an independent risk factor for death 1 year after surgery, and the mortality of hip fracture patients with lower weight was higher. This is consistent with the results of a large sample study conducted by K. Dig et al. [9, 20], who analyzed the Swedish national database of people over 65 years old who suffered a hip fracture between 2013 and 2016, classifying patients by weight according to four grades: underweight (< 22 kg/m^2^), normal weight (22–25 kg/m^2^), overweight (25–30 kg/m^2^), and obese (≧ 30 kg/m^2^). They found that mortality 1 year after surgery decreased as BMI classification increased. Taking normal weight as a reference, the risk of death was increased in patients with lower weight and decreased in patients with overweight and obesity. Thus, the “obesity paradox” appeared as an outcome variable for mortality 1 year after hip fracture surgery in our study. However, Akinleye et al. [3] evaluated data of patients represented in The National Surgical Quality Improvement Program database who underwent surgical procedures for hip fractures between 2008 and 2012, and they classified these patients into four grades: underweight (< 20 kg/m^2^), normal weight (20–29 kg/m^2^), obese (30–39 kg/m^2^), and morbidly obese (≧ 40 kg/m^2^). They found that underweight and morbidly obese patients had a significantly higher postoperative 30-day mortality rate than other patients, but the difference was not significant after controlling for confounding factors in the logistic regression. The differences between this study and ours may be due to the following reasons: (1) Difference in BMI classification; our classification was based on the principles stipulated by the World Health Organization, while this study was based on the willingness of the researchers. (2) Differences in included cases; our study included patients over 70 years old, but there was no age limit for the included patients in this study. (3) The inclusion range of patients' BMIs was different; in our included patients, there was no BMI over 40 kg/m^2^, so it was not possible to explore the difference in mortality risk between these patients and normal-weight patients. (4) Different follow-up nodes; we explored the 1-year mortality difference in BMI after hip fracture surgery, while this study explored the 30-day mortality difference in BMI after hip fracture surgery. Taken together, our results are convincing.

In addition, we found that after controlling for age, sex, CCI, surgery type and delay time of surgery, the risk of major complications was significantly higher in underweight patients and obese patients than in normal-weight patients (2.07 and 2.57 times, respectively). However, there was no significant difference in the risk of major complications between overweight and normal-weight patients. In addition, we found that the proportion of patients with major complications showed a U-shaped relationship with BMI, which was consistent with the results obtained by logistic regression after controlling for confounding factors. It is conceivable that underweight patients are at greater risk of postoperative malnutrition, and studies have shown that such postoperative malnutrition is strongly associated with adverse clinical outcomes, especially in older patients[4, 32]. For obese patients, obesity will cause a variety of chronic diseases to have an impact, affecting the development of clinical outcomes such as cardiac insufficiency, renal insufficiency, postoperative bleeding, postoperative infection, reoperation, readmission and prolonged length of stay. A study of first-time total hip replacements found that obese patients had an 8.2-fold increased risk of clinical complications compared with non-obese patients [35]. Y.P. Chaudhry et al. [9] evaluated the National Surgical Quality Improvement Program database for patients who underwent surgical procedures for hip fractures between 2008 and 2012. They classified these patients as underweight (< 18.5 kg/m^2^) or normal weight (18.5–24.9 kg/m^2^), overweight(25–29.9 kg/m^2^), and obese (30–39.9 kg/m^2^), morbidly obese (40–49.9 kg/m^2^), or superobese (≧ 50 kg/m^2^); they also found a U-shaped relationship between major and minor complications and BMI classification before and after controlling for confounding factors. However, there was a difference in the risk of major complications between obese patients and normal-weight patients in our study, while they found a difference in the risk of major complications between morbidly obese or superobese patients and normal-weight patients after controlling for confounding factors. These differences may be due to the different age ranges, races, and BMI classifications in the two studies.

In our study, we also explored the relationship between BMI and postoperative AKI. We found that the proportion of patients with AKI after hip fracture showed a U-shaped relationship among different BMIs. This is different from the study of Akinleye et al. [3], in which there was a linear increasing relationship between patients with postoperative AKI and BMI, possibly due to the different patients and BMI classifications included in the two studies. After controlling for confounding factors, underweight patients were found to have a significantly higher risk of AKI than normal-weight patients, which is consistent with the study by Ning Shi et al. and Hongran Moon et al. [21, 27]. In our study, we found that underweight patients had the most anemia before and after surgery, which may be the main reason for AKI in underweight patients. Perioperative blood transfusion was associated with BMI (*P < *0.05), and the perioperative blood transfusion rate was higher in patients with underweight. This is consistent with our findings on whether patients are preoperative anemia, the study on preoperative anemia in patients with hip fracture showed that preoperative anemia decreased linearly with the increase of BMI grades. Among them, the preoperative anemia rate was up to 50% in patients with underweight, which may be related to these patients as frail and having limited reserves to deal with a physiological stressful condition like hip fracture or the necessary surgery. Thus showing higher blood transfusion rates. In addition, in our study, there was also a correlation between anesthesia type and BMI (*P < *0.05), the proportion of general anesthesia patients in BMI classification showed a U-shaped relationship. Ying liang et al. showed that general anesthesia can increase the risk of postoperative AKI, which is closely related to the toxic effects of anesthetics on the kidney [18]. Toby et al. also showed that general anesthesia and perioperative blood transfusion were independently related to AKI [34], which was consistent with our research results. In addition, obese patients also seem to show an increased risk, consistent with studies by Wilfred Druml et al. and A B Pedersen et al. [11, 24]. An increasing number of studies have shown that obesity is associated with the occurrence of postoperative AKI, which can be explained by the following mechanisms. Studies have shown that increased systemic inflammation and oxidative stress are major features of obese and overweight patients, and increased levels of oxidative stress markers are closely related to AKI [6, 12, 30]. Intraoperative hypotension and postoperative bleeding can lead to decreased renal perfusion, decreased GFR, and increased serum creatinine, and achieving adequate renal perfusion may be more challenging in obese patients than in non-obese patients because hemodynamics are more difficult to measure in obese patients [8, 25, 28]. Because obese people are have a large amount of fat tissue and carry much water, it is difficult to recognize the presence of peripheral edema. In addition, the strong association between obesity and diabetes may explain why obese patients are more likely to develop AKI since damage to glomerular microvessels is an important complication of diabetes. In addition, obese patients are at higher risk of postoperative complications during hospitalization, such as bleeding, infection, and coagulopathy, which can cause AKI.

Finally, there are several limitations to our study. First, due to the research center, the number of patients included in this study was relatively small compared with most large studies. However, the results of our study were almost consistent with the results of most large studies, so our study results were more credible. Second, the follow-up time node was 1 year after the operation, but beyond this time node, the clinical outcome could not be obtained. Furthermore, the study could not be used to evaluate the skill of the surgeon, the integrity of the hospital’s auxiliary resources, and the reliability of nursing techniques, which may be associated with postoperative mortality or complications.

## Conclusion

This study demonstrated the association of BMI with 1-year mortality, major postoperative complications, and AKI. The 1-year mortality risk of patients with higher BMI was significantly reduced, while the 1-year mortality risk of patients with lower BMI was significantly increased. Compared with normal-weight patients, underweight patients and obese patients have a higher risk of major complications. Compared with normal-weight patients, underweight and obese patients have a higher risk of developing AKI. Therefore, for underweight patients, surgeons should pay more attention to perioperative blood volume changes, the selection of anesthesia methods and the dosage of various drugs during the perioperative period, so as to avoid AKI as far as possible. The relationship between BMI and these postoperative outcomes allows surgeons to formulate treatments based on the patient's BMI before surgery and to prevent possible adverse clinical outcomes, leading to better patient recovery.

## Data Availability

The datasets generated and analyzed during the current study are available from the corresponding author on reasonable request.
